# Network Analysis of Intrinsic Functional Brain Connectivity in Alzheimer's Disease

**DOI:** 10.1371/journal.pcbi.1000100

**Published:** 2008-06-27

**Authors:** Kaustubh Supekar, Vinod Menon, Daniel Rubin, Mark Musen, Michael D. Greicius

**Affiliations:** 1 Graduate Program in Biomedical Informatics, Stanford University School of Medicine, Stanford, California, United States of America; 2 Center for Biomedical Informatics Research, Stanford University School of Medicine, Stanford, California, United States of America; 3 Department of Psychiatry & Behavioral Sciences, Stanford University School of Medicine, Stanford, California, United States of America; 4 Program in Neuroscience, Stanford University School of Medicine, Stanford, California, United States of America; 5 Neuroscience Institute at Stanford, Stanford University School of Medicine, Stanford, California, United States of America; 6 Department of Radiology, Stanford University School of Medicine, Stanford, California, United States of America; 7 Department of Neurology and Neurological Sciences, Stanford University School of Medicine, Stanford, California, United States of America; Indiana University, United States of America

## Abstract

Functional brain networks detected in task-free (“resting-state”) functional magnetic resonance imaging (fMRI) have a small-world architecture that reflects a robust functional organization of the brain. Here, we examined whether this functional organization is disrupted in Alzheimer's disease (AD). Task-free fMRI data from 21 AD subjects and 18 age-matched controls were obtained. Wavelet analysis was applied to the fMRI data to compute frequency-dependent correlation matrices. Correlation matrices were thresholded to create 90-node undirected-graphs of functional brain networks. Small-world metrics (characteristic path length and clustering coefficient) were computed using graph analytical methods. In the low frequency interval 0.01 to 0.05 Hz, functional brain networks in controls showed small-world organization of brain activity, characterized by a high clustering coefficient and a low characteristic path length. In contrast, functional brain networks in AD showed loss of small-world properties, characterized by a significantly lower clustering coefficient (p<0.01), indicative of disrupted local connectivity. Clustering coefficients for the left and right hippocampus were significantly lower (p<0.01) in the AD group compared to the control group. Furthermore, the clustering coefficient distinguished AD participants from the controls with a sensitivity of 72% and specificity of 78%. Our study provides new evidence that there is disrupted organization of functional brain networks in AD. Small-world metrics can characterize the functional organization of the brain in AD, and our findings further suggest that these network measures may be useful as an imaging-based biomarker to distinguish AD from healthy aging.

## Author Summary

Alzheimer's disease (AD) is a brain disorder characterized by progressive impairment of episodic memory and other cognitive domains resulting in dementia and, ultimately, death. Functional neuroimaging studies have identified brain regions that show abnormal brain function in AD. Although there is converging evidence about the identity of these regions, it is not clear how this abnormality affects the functional organization of the whole brain. In order to characterize the functional organization of the brain, our approach uses small-world measures, which have also been used to study systems such as social networks and the internet. We use graph analytical methods to compute these measures of functional connectivity brain networks, which are derived from fMRI data obtained from healthy elderly controls and AD patients. The AD patients had significantly lower regional connectivity, and showed disrupted global functional organization, when compared to healthy controls. Moreover, our results indicate that cognitive decline in Alzheimer's disease patients is associated with disrupted functional connectivity in the entire brain. Our findings further suggest that small-world measures may be useful as an imaging-based biomarker to distinguish AD from healthy aging.

## Introduction

Alzheimer's disease (AD) is a neurodegenerative disorder characterized by progressive impairment of episodic memory and other cognitive domains resulting in dementia and, ultimately, death. Imaging studies in AD have begun a shift from studies of brain structure [1],[2] to more recent studies highlighting focal regions of abnormal brain function [3]–[6]. Most recently, fMRI studies have moved beyond focal activation abnormalities to dysfunctional brain connectivity.

Functional connectivity is defined as temporal correlations between spatially distinct brain regions [7]. PET studies, restricted to across-subject connectivity measures, have shown that AD patients have decreased hippocampus connectivity with prefrontal cortex [8] and posterior cingulate cortex [9] during memory tasks. Using fMRI, we demonstrated that AD patients performing a simple motor task had reduced intra-subject functional connectivity within a network of brain regions—termed the default-mode network—that includes posterior cingulate cortex, temporoparietal junction, and hippocampus [10]. Bokde et al. reported abnormalities in fusiform gyrus connectivity during a face-matching task in subjects with mild cognitive impairment—frequently a precursor to AD [11]. Three recent studies have reported reduced default-mode network deactivation in MCI and/or AD patients during encoding tasks [12],[13] and during a semantic classification task [14]. Celone et al also reported increased default-mode network deactivation in a subset of “less impaired” MCI patients.

In addition to analyzing functional connectivity during task performance, functional connectivity has also been investigated during task-free (“resting-state”) conditions. Task-free functional connectivity MRI detects interregional correlations in spontaneous blood oxygen level-dependent (BOLD) signal fluctuations [15]. Using this approach, Wang et al. found disrupted functional connectivity between hippocampus and several neocortical regions in AD [16]. Similarly, Li et al. reported reduced intrahippocampal connectivity during task-free conditions [17]. Most recently Sorg et al. [18] reported reduced resting-state functional connectivity in the default-mode network of MCI patients. Although evidence is accumulating that AD disrupts functional connections between brain regions [19], it is not clear whether AD disrupts *global* functional brain organization.

Graph metrics–the **clustering coefficient** and the **characteristic path length**—are useful measures of global organization of large-scale networks [20]. Graphs are data structures which have nodes and edges between the nodes. The clustering coefficient is a measure of local network connectivity. A network with a high average clustering coefficient is characterized by densely connected local clusters. The characteristic path length is a measure of how well connected a network is. A network with a low characteristic path length is characterized by short distances between any two nodes. Small-world network is characterized by a *high clustering coefficient* and a *low characteristic path length* [20],[21]. In a graphical representation of a brain network, a node corresponds to a brain region while an edge corresponds to the functional interaction between two brain regions. Functional connectivity networks of the human brain derived from electroencephalograms (EEGs), magnetoencephalograms and task-free fMRI data exhibit small-world characteristics [22]–[24]. In a recent EEG study, Stam et al. reported that small-world architecture in functional networks in the brain is disrupted in AD [25].

Here we examined the global functional organization of the brain in AD by (1) creating whole-brain functional connectivity networks from task-free fMRI data, (2) characterizing the organization of these networks using small-world metrics, and (3) comparing these characteristics between AD patients and age-matched controls. We hypothesized that global functional brain organization would be abnormal in AD. Further, given the need for a reliable, non-invasive clinical test for AD [26], we sought to determine whether a small-world metric obtained from task-free fMRI data might provide a sensitive and specific biomarker in AD.

## Results

### Subjects

Demographic data is shown in Table 1. Subject groups did not differ significantly in age (p = 0.73), gender distribution (p = 0.62), or years of education (p = 0.58). The mean MMSE was significantly lower (p<0.0001) for the AD group (22.14) compared to the controls (29).

**Table 1:** Subject Population–Demography and MMSE scores.

	AD (n = 21)	Controls (n = 18)
Age	63.97 (range: 48 to 83)	62.84 (range: 37 to 77)
Sex	10 males, 11 females	10 males, 8 females
Years of Education	15.89 (range: 12 to 22)	16.53 (range: 12 to 21)
MMSE	22.14* (range: 12 to 29)	29* (range: 27 to 30)

MMSE scores are significantly different in AD patients compared with control subjects (*denotes significant differences between groups).

### Analyses of small-world metrics at different scales

**Figure 1:**
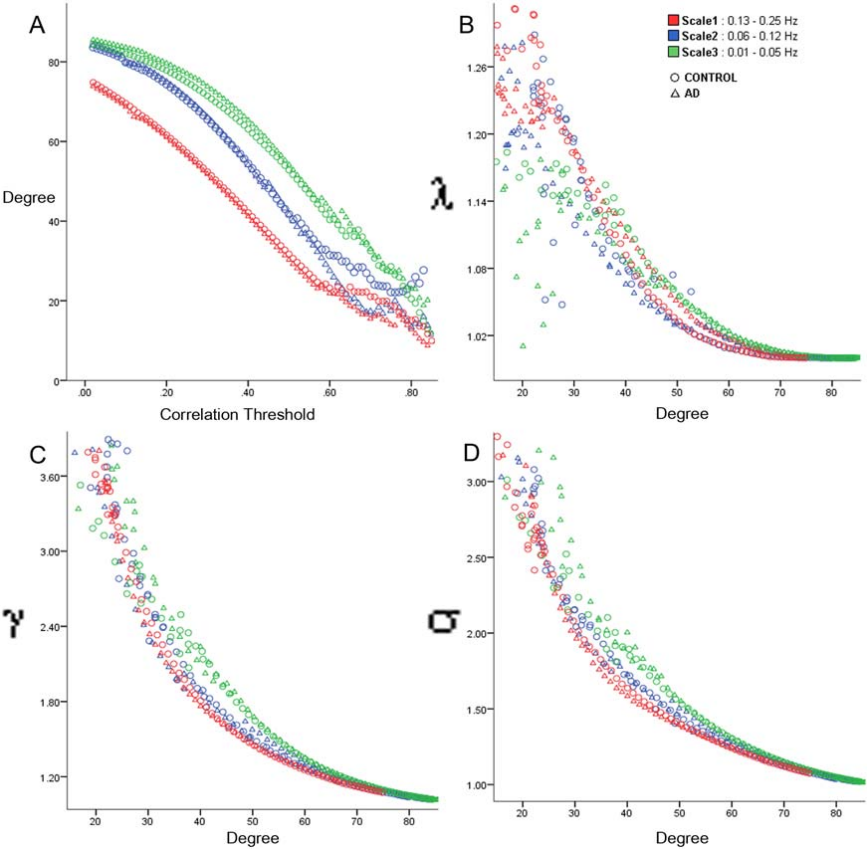
Graph metrics–degree, λ (L/L_ran_), γ (C/C_ran_), σ (γ/λ), for the AD group (Δ) and the control group (○) at three frequency intervals–0.01 to 005 Hz (green), 0.06 to 0.12 Hz (blue), and 0.13 to 0.25 Hz (red). (A) For both groups, the mean degree–a measure of network connectivity is highest at Scale 3 for a wide range of correlation thresholds (0.01<R<0.7), (B) The mean characteristic path length (λ) is low (1<λ<1.27) and shows similar trends at all the scales (C) The clustering coefficient (γ) for both groups is highest at Scale 3. (D) Due to higher mean γ values, the small-world measure σ (γ/λ) is highest at Scale 3 for both groups. The small-world property (σ>1) showed a linear increase in small-worldness as the threshold increased (degree decreased). σ values for higher correlation thresholds are hard to interpret as at higher threshold values graphs of functional brain networks have fewer edges (smaller degree) and tend to split into isolated sub-graphs.

We first examined graph metrics obtained for the functional brain networks constructed by thresholding (threshold values ranged from 0.01 to 0.99 with an increment of 0.01) the wavelet correlation matrix computed at three scales (frequencies in the range from 0.01 to 0.25 Hz) for the AD group and the control group (see Figure 1). For both groups, the mean degree was highest at Scale 3 for a wide range of correlation thresholds (0.01<R<0.7). The mean characteristic path length (λ) for both groups, when controlled for the degree of the network, was low (1<λ<1.27) and showed similar trends at all the scales. The clustering coefficient (γ) for both groups, when controlled for the degree of the network, was highest at Scale 3. Due to higher mean γ values, the small-world measure σ (γ/λ), when controlled for degree of the network, was highest at Scale 3 for both groups. The small-world property (σ>1) showed a linear increase in small-worldness as the threshold increased (degree decreased). σ values for higher correlation thresholds are difficult to interpret, as at higher threshold values, graphs of functional brain networks have fewer edges (smaller degree) and tend to split into isolated sub-graphs. Graph metrics such as clustering coefficient, characteristic path length, and small-world property do not meaningfully characterize network structures that are not composed of a single, large group of interconnected nodes [20].

Since functional connectivity and small-world properties were salient at lower-frequencies (0.01 to 0.05 Hz) for the AD group and the control group, we only report results for this frequency interval in subsequent analyses.

### Comparison of small-world metrics in the AD and control groups

**Figure 2:**
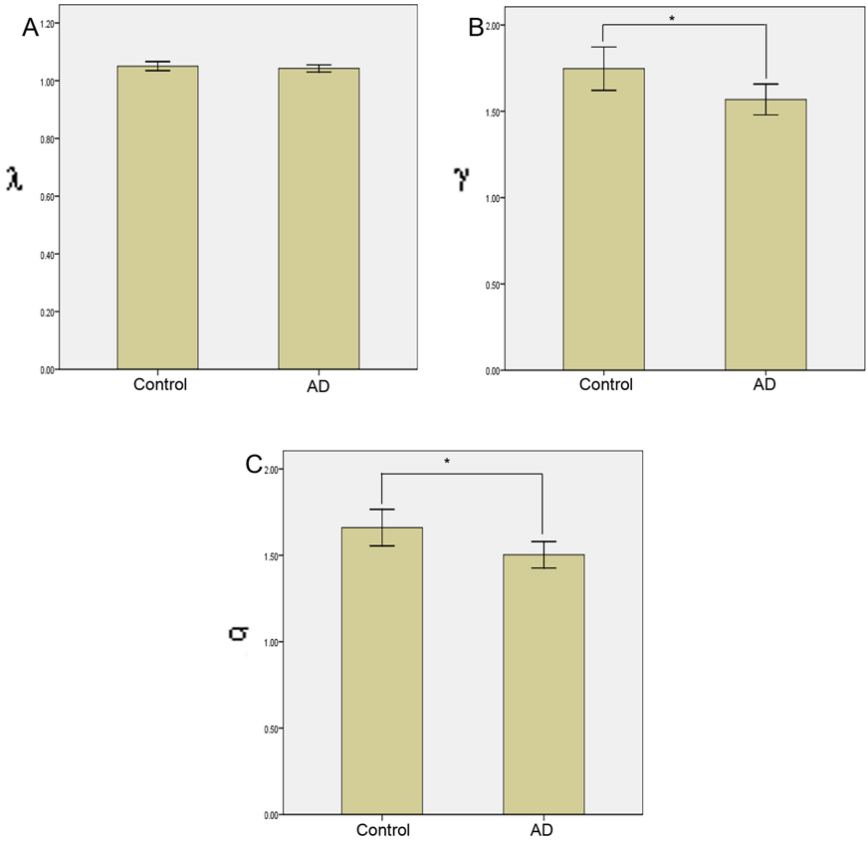
Small-world properties for networks derived by thresholding the correlation matrices such that the network has K′ edges. Error bars represent values two standard deviations from the mean. (A) Mean λ (L/L_ran_) values for the AD group and the control group. No significant differences in the mean λ values are observed. (B) Mean γ (C/C_ran_) values for the AD group and the control group. γ values in AD group were significantly lower (indicated by *) than that in the control group (p<0.01). (C) Mean σ (γ/λ) values for the AD group and the control group. σ values in AD group were significantly lower (indicated by *) than that in the control group (p<0.01).

In the frequency interval between 0.01 to 0.05 Hz, we examined λ and γ values in the two groups. For group comparison, we controlled for the average correlation value (r). r is different across groups. Thus, for a given correlation threshold, the number of edges in the graph are likely to be less in AD, resulting in high λ and low γ values. To ensure that graphs in both groups had the same number of edges, individual correlation matrices were thresholded such that the resultant graph had exactly K′ edges. K′ is the average number of edges in the graph obtained by thresholding individual correlation matrices with R = r_i_ (r_i_ is the average correlation value for subject i, i = 1 to 39). The value of K′ selected according to this procedure was 40 for both the groups. Mean λ, mean γ, and mean σ values for the networks of the AD group and control group were derived by thresholding the correlation matrices such that the network has K′ ( = 40) edges (shown in Figure 2). Results were: (i) No significant differences in the mean λ values were observed, Mean γ values in the AD group were significantly lower than in the control group (p<0.01), and (iii) Mean σ values in the AD group were significantly lower than in the control group (p<0.01).

### Analysis of global efficiency of whole-brain functional connectivity network

**Figure 3:**
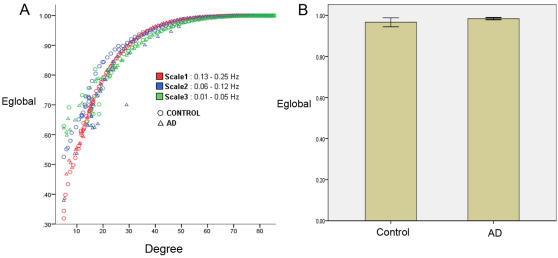
Global efficiency of whole-brain functional connectivity network. (A) Global efficiency measure (E_global_), for the AD group (Δ) and the control group (○) at three frequency intervals–0.01 to 005 Hz (green), 0.06 to 0.12 Hz (blue), and 0.13 to 0.25 Hz (red). The mean E_global_ value is low (0.78<λ<1) and shows similar trends at all the scales. (B) For the frequency interval 0.01 to 005 Hz (green)–mean E_global_ values for the AD group and the control group for networks derived by thresholding the correlation matrices such that the network has K′ edges. No significant differences in the mean E_global_ values were observed. Error bars represent values two standard deviations from the mean.

We examined global efficiency (E_global_) values obtained for the functional brain networks constructed by thresholding (threshold values ranged from 0.01 to 0.99 with an increment of 0.01) the wavelet correlation matrix computed at three scales (frequencies in the range from 0.01 to 0.25 Hz) for the AD group and the control group (see Figure 3A). The mean E_global_ for both groups, when controlled for the degree of the network, was low (0.77<E_global_<1) and showed similar trends at all the scales.

In the frequency interval 0.01 to 0.05 Hz (scale 3), mean E_global_ values for the AD group and the control group for the networks derived by thresholding the correlation matrices such that the network has K′ ( = 40) edges are shown in Figure 3B. No significant differences in the mean E_global_ values were observed.

### Specificity and sensitivity of clustering coefficient in distinguishing AD participants from controls

**Figure 4:**
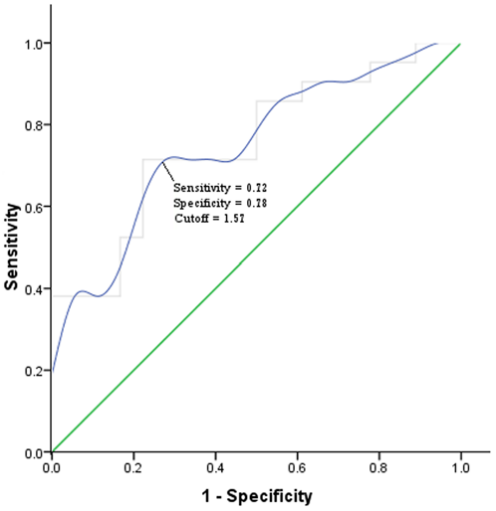
Receiver operating characteristic curve, plot of the sensitivity vs. (1-specificity) for distinguishing AD participants from controls as a function of varying normalized clustering coefficient (γ) threshold. Using a cut-off value of 1.57, γ correctly classified 14 out of 18 controls and 15 of 21 AD subjects yielding 72% sensitivity and 78% specificity. The Area under the curve was 0.754 (95% CI Area 0.602 to 0.906).

Here, we examined whether γ (normalized clustering coefficient) might prove sufficiently sensitive and specific to serve as a biomarker for AD. Using the cut-off value (γ = 1.57) that maximizes sensitivity and specificity, γ correctly classified 14 out of 18 controls and 15 of 21 AD subjects, yielding 72% sensitivity and 78% specificity respectively. A receiver operating characteristic curve for various cut-off values is shown in Figure 4. The Area Under the Curve for the ROC was 0.754 (95% CI Area 0.602 to 0.906).

### Regional profile of clustering coefficient

**Figure 5:**
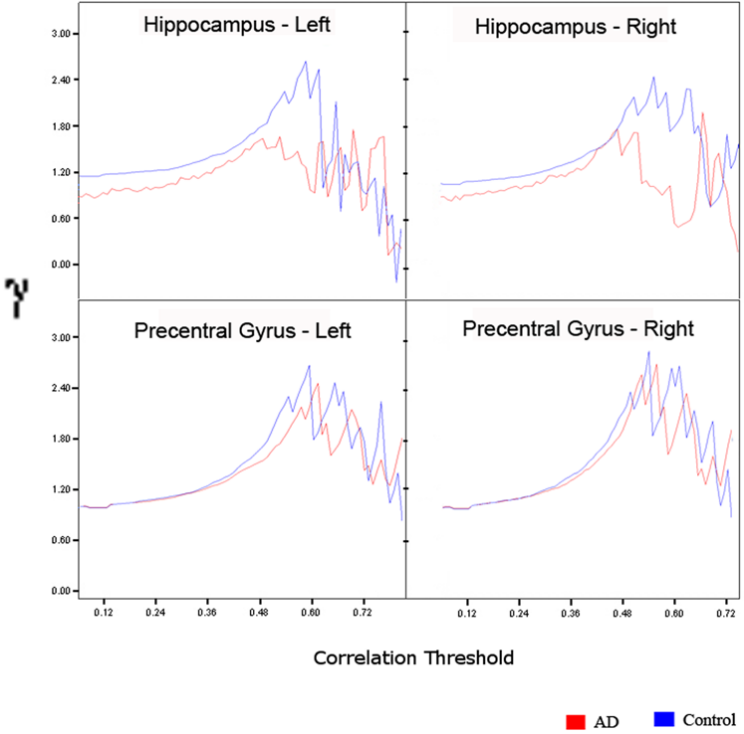
Small-world property γ (C/C_ran_), the normalized clustering coefficient, for four regions of interest–left hippocampus (Hippocampus - Left), right hippocampus (Hippocampus - Right), left precentral gyrus (Precentral Gyrus - Left), right precentral gyrus (Precentral Gyrus - Right)–for the AD group (red) and the control group (blue) as a function of the correlation threshold. In the left and the right hippocampus, for threshold values from 0.1 to 0.6, the clustering coefficient values in the AD group were significantly lower (p<0.01) than in the control group, while for the left and the right precentral gyrus, no significant differences in the clustering coefficient values were observed at any correlation threshold.

Figure 5 shows a plot of γ for each of the four regions, for the AD group and the control group as a function of the correlation threshold. In the left and the right hippocampus, the fitted growth curve was significantly lower (p<0.01) in the AD group, compared to the control group, reflecting lower clustering coefficient values for a range of threshold values from 0.1 to 0.6. A similar analysis in the left and right precentral gyrus, revealed no significant differences in the clustering coefficient values. Across the four regions, no significant differences in the clustering coefficient values were observed for correlation threshold values >0.6, mainly due to the large variance observed at higher threshold values. This analysis was extended to the remaining 86 regions of the whole brain functional network (see Table S1 to find regions that showed significant differences in clustering coefficient values between the two subject groups).

To determine whether the differences observed in γ values reflect true differences and not artifacts of different average correlation values, we repeated our analysis by computing γ values as a function of the number of edges in the graph. Mean γ values of four anatomical regions of interest for the AD group and the control group for networks derived by thresholding the individual correlation matrices such that the network has K′ edges were computed and compared. Results were consistent with the initial analysis–significantly lower clustering coefficient values (p<0.01) in the left and right hippocampus in AD, and no significant differences in the left and right precentral gyrus.

### Regional connectivity

We next examined regional correlation values (connectivity) in the two groups. Results show that compared to the control group, the AD group had decreased correlation values (1) within the temporal lobe, (2) between the temporal lobe and thalamus, (3) between the temporal lobe and corpus striatum, (4) between the thalamus and occipital lobe, and (5) between the thalamus and other parts of the frontal lobe, but increased correlations (1) within the prefrontal areas, (2) within other parts of frontal lobe, (3) between the prefrontal areas and other parts of the frontal lobe, and (4) between other parts of frontal lobe and the corpus striatum.

### Reproducibility of findings

To determine if our findings were robust–reproducible across datasets–we repeated our entire analysis on a second resting-state fMRI dataset (rest2 scans) acquired from the same set of subjects. Results were consistent with previous analysis (performed on data from rest1 scan): (i) Functional brain connectivity and small-world metrics including the global efficiency were salient in the low frequency interval–0.01 to 0.05 Hz (Scale 3), (ii)No significant differences in the mean λ values were observed, (iii) Mean γ values in the AD group were significantly lower than in the control group (p<0.01), (iv) Mean σ values in the AD group were significantly lower than that in the control group (p<0.01), (v) No significant differences in the mean E_global_ values were observed, and (vi) significantly lower clustering coefficient values were found in the left and right hippocampus in AD, with no significant differences in the left and right precentral gyrus.

## Discussion

In this study, we investigated whether global functional brain organization is disrupted in AD. To our knowledge, this is the first study to examine alterations in global functional organization and connectivity in AD patients using fMRI data. Graph metrics–clustering coefficient and characteristic path length—were used to measure and characterize global functional organization in the brain. The main finding of our study is that functional brain networks in AD consistently showed lower clustering but similar characteristic path lengths compared to controls, which suggests disrupted global functional organization in AD. Our findings also suggest that small-world network characteristics might be useful as an imaging biomarker for AD.

The characteristic path lengths were low (λ∼1) and showed no significant differences between the AD group and the control group, suggesting short distances between distinct brain regions in both groups. This finding suggests an organization consisting of multiple short alternative paths between nodes in functional brain networks in both groups.

The most interesting finding of our study was the lower levels of clustering observed in the AD group. Clustering coefficient is a measure of local efficiency or the fault-tolerance of a network [21]. The difference in clustering coefficients in the AD group as compared to the control group was observed at a correlation threshold at or near a subject's average correlation (to ensure an equivalent number of edges across subjects), and the clustering coefficient was significantly lower in the AD group, suggesting loss of local efficiency in AD. Similarly, values for σ, a measure of small-worldness, were significantly lower in the AD group compared to the control group, suggesting loss of small-world properties in AD.

Analysis of global efficiency in functional brain networks showed that the networks exhibit small-world properties indicated by smaller E_global_ values compared to random networks, but this measure was not significantly different. This finding parallels results obtained with measures of characteristic path length.

Regional analysis of differences in clustering coefficients as a function of correlation thresholds showed that the left and the right hippocampal regions differed significantly between groups. In contrast, the clustering coefficient of the precentral gyrus did not differ between groups. This suggests disrupted connectivity from the hippocampus to other regions of the brain in AD. This finding is consistent with our previous study [10] showing that AD reduced functional connectivity of the hippocampus within a specific network of regions—the default mode network [27],[28] that includes the posterior cingulate and lateral temporoparietal cortices. It is also consistent with the study by Wang et al. [16] showing altered hippocampal connectivity to several neocortical regions in the early stages of AD. Other studies have reported decreased intrahippocampal synchrony of low frequency BOLD fluctuations [17] during a task-free scan. Taken together, these findings point to significantly altered local and global hippocampal network connectivity in AD.

Analysis of the group differences in the regional connectivity across several broadly defined anatomical regions demonstrate that AD patients not only showed decreased intratemporal, temporo-thalamus, temporo-corpus striatum, thalamo-occipital and thalamo-frontal connectivity but, surprisingly, also showed increased intrafrontal, frontal-prefrontal, and fronto-corpus striatum connectivity. These findings are in line with the recent study by Wang et al. [29] which not only reported decreased connectivity between a number of regions, but also increased prefrontal connectivity in AD. As suggested by fMRI studies showing increased prefrontal activation in AD during task performance [30], these findings suggest that patients with AD may rely on increased prefrontal connectivity to compensate for reduced temporal connectivity. An intriguing (and testable) hypothesis is that the ability to make such compensatory changes in frontal lobe connectivity may account in part for the “cognitive reserve” phenomenon [31] that allows some patients to perform better than others despite equivalent pathological burdens.

Small-world characterization is well-suited for analyzing anatomical and functional brain networks at the system level because these networks are complex and optimally connected to minimize information processing costs [32],[33]. Anatomical connectivity networks of the brain obtained from tracer studies in the primate cortical visual system [34], primate cerebral cortex [35], and macaque cortex [36] have been shown to exhibit small-world characteristics. Functional connectivity networks of human brain constructed from EEG as well as MEG data have also been shown to have small-world architecture [22],[23]. Salvador et al. [37] built a whole-brain functional connectivity network from task-free human functional MRI data. This network of intrinsic, task-free functional interactions between 90 cortical regions was also shown to have small-world properties–high clustering coefficient and low characteristic path length. The small-world architecture was confirmed by Achard et al., who also reported that the small-world properties were salient in the frequency interval 0.03 to 0.06 Hz [24],[32]. These findings suggest that the structural and functional organization of the brain has a small-world architecture; these characteristics may assist in robust and dynamic information processing. Recently, Stam et al [25]. reported that the architecture of whole-brain functional networks derived using scalp EEG is disrupted in AD. They observed that a 21-node network constructed using EEG data collected from subjects with AD showed loss of small-world properties characterized by longer characteristic path length with relative sparing of the local clustering.

Table 2 provides a comparison of results obtained from our study to all of the above-mentioned results on the small-world characterization of functional brain networks. Our results are largely comparable to small-world metrics reported by Salvador et al. also using task-free fMRI in healthy human subjects [24],[37]. The small-world metrics reported by Stam et al. analyzing beta-band EEG in controls and AD subjects are also largely consistent with our results [25]. It is interesting to note that whereas we report similar characteristic path lengths but different cluster coefficients between AD and controls, the EEG study found the converse (characteristic path lengths differed between AD subjects and controls but cluster coefficients did not). We believe that this discrepancy may be related to significant volume conduction in scalp EEG data [38] which may reduce sensitivity to detect differences in short-range connectivity while enhancing the relative sensitivity to detect differences in long-range connectivity. Other methodological differences may also contribute–the use of synchronization likelihood as their association measure, which unlike wavelet correlation is sensitive to non-linear coupling. Also, the poor spatial resolution of scalp EEG limits the network to mainly cortical regions, unlike our fMRI study where the network comprised of cortical as well as sub-cortical regions, which is a relative strength of our study.

**Table 2:** Comparison of number of nodes in the graph (N), normalized characteristic path length (λ), normalized clustering coefficient (γ), and small-world measure (σ) from our study with previously published results on small-world characterization of functional brain network constructed using EEG, MEG, and fMRI data.

Data	N	λ	γ	σ
fMRI of healthy human subjects (Our study)	90	1.05	1.74	1.66
fMRI of human subjects with AD (Our study)	90	1.042	1.56	1.497
EEG of healthy human subjects (Stam 2006)	21	1.07	1.58	1.476
EEG of human subjects with AD (Stam 2006)	21	1.12	1.6	1.428
EEG of healthy human subjects (Micheloyannis 2006)	28	1.0	2.0	2.0
MEG of healthy human subjects (Stam 2004)	126	1.8	4.2	2.3
fMRI of healthy human subjects (Salvador 2005)	90	1.09	2.08	1.91
fMRI of healthy human subjects (Achard 2006)	90	1.09	2.37	2.18

To address the extent to which clustering coefficients serve as a sensitive biomarker to distinguish AD from healthy aging, we examined γ values in the two subject groups. The clustering coefficient is a measure of efficiency in network connectivity. It distinguished AD subjects from controls with a sensitivity of 72% and specificity of 78%. These values approach the sensitivity and specificity reported for other imaging biomarkers [10], [39]–[41] and are close to the range considered clinically relevant by a recent Working Group on biomarkers in AD [42]. With some improvements in the technique—decreasing the number of nodes in the network for example—the clustering coefficient may therefore prove to be an effective biomarker for AD, though prospective studies will be required to validate its effectiveness. In addition to its promise as a diagnostic aid, the clustering coefficient merits investigation as a functional marker of response to treatment.

This study has two main limitations. First, in evaluating its efficacy as a biomarker, it will be critical to assess this metric not only in AD and normal subjects, but in subjects with non-AD dementias and related conditions to ensure that these findings are specific to AD and not to dementia or other neurodegenerative disorders more generally. The second limitation pertains to the fact that most of the AD patients (14 of 21), and none of the controls, were taking an acetylcholinesterase inhibitor. Similarly, 12 of 21 AD patients, and none of the controls, were taking memantine, an NMDA-receptor antagonist. While we doubt that these differences in medication exposure could account for the differences in clustering coefficients in AD subjects we cannot exclude that possibility in the current study.

In conclusion, we have demonstrated that fMRI-derived functional brain networks in AD show loss of small-world properties. Our findings suggest that cognitive decline in AD is associated with disrupted global functional organization in the brain.

## Materials and Methods

### Participants

Twenty one subjects with AD and eighteen age-matched control subjects participated in this study after giving written, informed consent. For those AD patients who were unable to give informed consent, written, informed consent was obtained from their legal guardian. The study protocol was approved by the Stanford University Institutional Review Board. The AD subjects (10 males, 11 females) ranged in age from 48 to 83 (mean age 63.97) with 12 to 22 years of education (mean years of education 15.89). The subjects were recruited from memory disorder clinics in Stanford University and the University of California San Francisco (UCSF). All AD subjects met the NINDS-ADRDA criteria for probable AD [43]. One subject had a presenilin-1 mutation; a second subject's mother had a presenilin-2 mutation (the subject herself did not wish to be tested). Diagnosis of three other subjects has since been confirmed at autopsy. ApoE status was known for 4 additional AD subjects: one was homozygous for the ApoE 4 allele and 3 were heterozygous for the ApoE 4 allele. The control subjects (10 males, 8 females) ranged in age from 37 to 77 (mean age 62.84) with 12 to 21 years of education (mean years of education 16.53). Study subjects were recruited from several sources (partners of AD patients, participants in a longitudinal study of normal aging at UCSF, and Stanford research staff). Control subjects denied any significant neuropsychiatric disease or memory trouble, were not taking any psychoactive medicines, and had to have a Mini Mental State Examination (MMSE) score of 27 or more. 14 of 21 AD patients were taking an acetylcholinesterase inhibitor. And, 12 of 21 AD patients were taking memantine, an NMDA-receptor antagonist. The MMSE score of the AD group ranged from 12 to 29 (mean MMSE score 22.14) and the MMSE score of the control group ranged from 27 to 30 (mean MMSE score 29). Each subject underwent an MMSE, a structural MRI scan, and a task-free fMRI scan.

### Data acquisition

For the task-free scan, subjects were instructed to keep their eyes closed and try not to move. The scan lasted for 6 minutes (rest1 scan). All the subjects (except for one control subject and two AD subjects) also underwent another task-free scan that lasted for 6 minutes (rest2 scan) and was acquired immediately after the first task-free scan. Functional images were acquired on a 3-T General Electric Signa scanner using a standard whole-head coil. Twenty-eight axial slices (4 mm thick, 1mm skip) were acquired parallel to the plane connecting the anterior and posterior commissures and covering the whole brain using a T2* weighted gradient echo spiral in/out pulse sequence (TR = 2000 msec, TE = 30 msec, flip angle = 80° and 1 interleave) [44]. To aid in the localization of functional data, a high resolution T1-weighted spoiled grass gradient recalled (SPGR) 3D MRI sequence with the following parameters was used: 124 coronal slices 1.5 mm thickness, no skip, TR = 11 ms, TE = 2 ms, and flip angle = 15°.

### Data preprocessing

Data (rest1 and rest2 scans) were preprocessed using statistical parametric mapping (SPM2) software (http://fil.ion.ucl.ac.uk/spm). The first 8 image acquisitions of the task-free functional time series were discarded to allow for stabilization of the MR signal. Each of the remaining 172 volumes underwent the following preprocessing steps: realignment, normalization, and smoothing. Normalization was to the Montreal Neurological Institute (MNI) template and smoothing was done with a 4 mm full width half maximum Gaussian kernel to decrease spatial noise. Excessive motion, defined in our lab as greater than 3.5 mm of translation or 3.5 degrees of rotation in any plane, was not present in any of the task-free scans.

### Anatomical parcellation

The preprocessed task-free functional MRI datasets were parcellated into 90 regions using anatomical templates defined by Tzourio-Mazoyer et al. [45]. A task-free fMRI timeseries was computed for each of the 90 regions by averaging all voxels within each region at each time point in the time series, resulting in 172 time points for each of the 90 anatomical regions of interest. These regional fMRI time series were then used to construct a 90 node whole-brain task-free functional connectivity network for each subject.

### Construction of whole-brain functional connectivity network

Wavelet analysis was used to construct correlation matrices from the regional fMRI time series data. These matrices described frequency-dependent correlations, a measure of functional connectivity, between spatially-distinct brain regions. Correlation matrices were then thresholded to generate a whole-brain functional connectivity network.

Wavelets are mathematical functions that transform the input signal into different frequency components [46]. Wavelets are appropriate methods for the analysis of task-based as well as task-free fMRI signal [24],[47]. In our study, we applied a maximum overlap discrete wavelet transform (MODWT) to each of the 90 regional time series from each subject to obtain the contributing signal in the following three frequency components: scale 1 (0.13 to 0.25 Hz), scale 2 (0.06 to 0.12 Hz), and scale 3 (0.01 to 0.05 Hz). To account for a relatively small number (172) of data points per time series for low frequency correlation analysis, the vector representing the time series beyond its boundaries (<0 and >172) was assumed to be a symmetric reflection of itself. At each of the three scales, wavelet correlations between signals in the 90 anatomical regions were determined by computing the correlation coefficient between the transformed signals at that scale.

For each subject, a 90-node, scale-specific, undirected graph of the functional connectivity network was constructed by thresholding the wavelet correlation matrix computed at that scale. If the wavelet correlation value between two anatomical regions represented by nodes i and j in the network exceeded a threshold then an edge was drawn between node i and node j. There is currently no formal consensus regarding threshold selection, so we computed networks for threshold values from 0.01 to 0.99 with an increment of 0.01. Once a whole-brain functional connectivity network was constructed from the correlation matrix, we characterized this network in terms of its small-world properties.

### Small-world analysis of the whole-brain functional connectivity network

Small-World properties of a network are described by the clustering coefficient and the characteristic path length of the network. The clustering coefficient and characteristic path length of functional brain networks generated from the task-free fMRI data obtained from 21 AD subjects and 18 age-matched controls were computed. The clustering coefficient of every node was computed as the ratio of the number of connections between its neighbors divided by the maximum possible connections between its neighbors. The clustering coefficient (C) of the network was calculated as the mean of the clustering coefficients of all the nodes in the network. The mean minimum path length of a node was computed as the average of minimum distances from that node to all the remaining nodes in the network. The characteristic path length (L) of the network was the average of the mean minimum path lengths of all the nodes in the network. The clustering coefficient and path length of nodes completely disconnected with the network were set as 0 and Inf respectively, and these nodes were excluded while computing C and L. To evaluate the network for small-world properties, we compared the clustering coefficient and the characteristic path length of the network with corresponding values (C_ran_, L_ran_) obtained and averaged across 1000 random networks with the same number of nodes and degree distribution [48]. Degree of a network is a measure of its connectivity. The degree of every node was computed by counting the number of edges incident on that node. Small world networks are characterized by high normalized clustering coefficient γ (C/C_ran_)>1 and low normalized characteristic path length λ (L/L_ran_)∼1 compared to random networks [24]. A cumulative metric σ–the ratio of normalized clustering coefficient (γ) to the characteristic path length (λ), a measure of small-worldness–is thus greater than 1 for small world networks.

### Analysis of global efficiency of whole-brain functional connectivity network

Small-world networks are characterized by high clustering coefficient and low characteristic path length. These small-world metrics, particularly the path length, are not meaningful when the graph contains disconnected nodes. To address this issue, we ensured that only small-world metrics computed on connected graphs were considered in our analysis. Specifically, the algorithm used to choose the correlation threshold (R) guaranteed that disconnected graphs were excluded from the analysis. Also, in the node-wise clustering coefficient comparison analysis, we only considered thresholds from 0.1 to 0.6. We chose these thresholds because beyond 0.6 the network gets divided into disconnected subset of nodes.

To determine if our characteristic path length findings were robust and reliable, we computed efficiency of functional brain networks. It has been previously reported that efficiency as a graph metric (1) is not susceptible to disconnected nodes, (2) is applicable to unweighted as well as weighted graphs, and (3) is a more meaningful measure of parallel information processing than path length [49]. Efficiency of a graph (E_global-net_) [50] is inverse of the harmonic mean of the minimum path length between each pair of nodes, L_ij_, and was computed as,
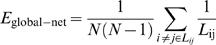
(1)


To evaluate the network for its global efficiency of parallel information processing, we compared the global efficiency of the network (E_global-net_) with corresponding values (E_global-ran_) obtained and averaged across 1000 random networks with the same number of nodes and degree distribution. A network with small-world properties is characterized by global efficiency value that is lower than the random network–E_global_ (E_global-net_/E_global-ran_)<1.

### Regional profile of clustering coefficient

In the frequency interval 0.01 to 0.05 Hz, we next examined small world metric values of four anatomical regions of interest in the two groups. These four regions included the left hippocampus, the right hippocampus, the left precentral gyrus, and the right precentral gyrus. These were chosen because we hypothesized significant differences in the hippocampus (a region targeted early in AD), but not in the precentral gyrus (which is typically spared even in the advanced stages of AD) [51]. This regional profiling analysis was performed on the clustering coefficient (and not the path length) because only the former differed significantly between the AD and control groups.

Growth curve modeling, with an intercept (baseline), linear and quadratic terms, was used to compare the clustering coefficient values for threshold values from 0.1 to 0.6 in the two subject groups. We chose these thresholds because beyond 0.6 the network divides into disconnected subsets of nodes and small-world metrics are then no longer meaningful [20]. This analysis was performed using the Mplus software (http://www.statmodel.com). Growth curve models describe change (growth) with respect to a control variable. They are well-suited for analyzing group-level differences in biomedical data, particularly in cases where capturing and analyzing individual growth trajectories is important. In our study, the growth trajectories of clustering coefficient of a subject carry important information about the variance within the group and needs to be incorporated in the model. The coefficients of growth curve models capture the baseline performance, instantaneous growth rate, and the acceleration of the variable of interest–γ.

### Regional connectivity

We then examined regional correlation values (connectivity) in the two groups. Wavelet correlation values of 4005 pairs of anatomical regions were first z-normalized and then compared between the two subject groups. T-test with a false discovery rate of 0.01 was used to test if the difference was significant. For the frequency range 0.01 to 0.05 Hz, the correlation values of 108 pairs of anatomical regions out of a total 4005 pairs were significantly lower in the AD group as compared to the control group while only 42 correlation values showed a significant increase in the AD group (p<0.01, corrected for multiple comparisons). To get an idea of average differences in the global functional organization in the two groups, we investigated the regional connectivity at a coarser level of granularity. Ninety anatomical regions of our network were grouped into eight higher-level anatomical regions using the grouping defined by Tzourio-Mazoyer et al. [45]. The *prefrontal lobe region* consists of the superior frontal gyrus (dorsolateral, orbital, medial, medial orbital), the middle frontal gyrus, the middle frontal gyrus (orbital), the inferior frontal gyrus (opercular, triangular, orbital), the olfactory gyrus, the gyrus rectus, and the anterior cingulate. The *other parts of frontal lobe region* consists of the precentral gyrus, the supplementary motor area, the median cingulate, and the rolandic operculum. The *occipital lobe region* consists of the calcarine fissure, the cuneus, the lingual gyrus, the superior occipital gyrus, the middle occipital gyrus, and the inferior occipital gyrus. The *temporal lobe and the medial temporal region* consists of the superior temporal gyrus, the temporal pole (superior, middle), the middle temporal gyrus, the inferior temporal gyrus, the heschl gyrus, the fusiform gyrus, the hippocampus, the parahippocampal gyrus, and the amygdala. The *parietal lobe region* consists of the postcentral gyrus, the superior parietal lobule, the inferior parietal lobule, the supramarginal gyrus, the angular gyrus, the precuneus, the paracentral lobule, and the posterior cingulate gyrus. The *corpus striatum region* consists of the caudate nucleus, the putamen, and the pallidum. Each higher level anatomical region consists of regions from both the hemispheres. Differences in mean correlation coefficients for 4005 pairs were aggregated into 32 pairs and the resulting differences were then normalized. (see also [52]). In the aggregation step, the number of decreased (−1) or increased connectivities (+1) for each of the 32 pairs ( = (8×8)/2) was counted. For example, to identify differential connectivity between the *prefrontal lobe region* and the *occipital lobe region* the number of decreased or increased connectivities between all pairs of sub-regions belonging to the *prefrontal lobe region* and *occipital lobe region* was counted. Since each brain region has a different number of sub-regions, the aggregated differential connectivity count was normalized by the number of possible connections between pairs of sub regions belonging to the two brain regions under investigation.

## Supporting Information

Table S1 Regions of whole brain functional network ranked in ascending order of the p-value (computed using growth curve modeling) and then descending order of absolute difference between the clustering coefficient values of the AD group and the control group.
(0.18 MB DOC)
