# Supplementary material for: Network Analysis of Intrinsic Functional Brain Connectivity in Alzheimer's Disease
Source: PLoS Comput Biol. 2008 Jun 27;4(6):e1000100. doi: 10.1371/journal.pcbi.1000100 (PMC2435273; doi:10.1371/journal.pcbi.1000100)
Supplement: Table S1 — Regions of whole brain functional network ranked in ascending order of the p-value (computed using growth curve modeling) and then descending order of absolute difference between the clustering coefficient values of the AD group and the control group. (0.18 MB DOC) [file pcbi.1000100.s001.doc]

**Table S1. Regions of whole brain functional network ranked in ascending order of the p-value (computed using growth curve modeling) and then descending order of absolute difference between the clustering coefficient values of the AD group and the control group.** γ_i_, λ_i_, Eglobal_i_, and K_i_ are normalized clustering coefficient, normalized path length, normalized efficiency, and degree, respectively, of the ith region. The regions are classified as subcortical or primary, association, limbic, or paralimbic cortex. Regions with significant differences (p < 0.01) in the clustering coefficient values are listed in italics. The p-value was computed by comparing the fitted growth curve of the AD group with the control group, of clustering coefficient values for a range of threshold values from 0.1 to 0.6. The γ_i_  values reported in the table are for the correlation threshold that generates a network with exactly K’ edges. This may explain the discrepancy – some regions such as the right Anterior Cingulate Gyrus show measurable difference in γ_i_ (Control group = 0.98 vs AD group = 1.56) but were found to be not significant (using growth curve modeling which considers range of clustering coefficient values)

| Brain Region | Class | **γ_i_** | | **λ_i_** | | Eglobal_i_ | | K_i_ | |
| --- | --- | --- | --- | --- | --- | --- | --- | --- | --- |
|  |  | *Control* | *AD* | *Control* | *AD* | *Control* | *AD* | *Control* | *AD* |
| *Paracentral lobule, Left* | *Association* | *1.84* | *1.33* | 1.00 | 0.99 | 1.01 | 1.05 | 52.24 | 42.17 |
| *Posterior Cingulate Gyrus, Left* | *Paralimbic* | *1.89* | *1.46* | 1.10 | 1.15 | 0.92 | 0.90 | 32.62 | 31.61 |
| *Heschl's gyrus, Right* | *Primary* | *1.74* | *1.33* | 1.14 | 1.12 | 0.77 | 0.82 | 27.38 | 18.00 |
| *Inferior parietal lobule, Right* | *Association* | *1.90* | *1.53* | 1.09 | 1.02 | 0.92 | 1.00 | 45.00 | 31.11 |
| *Superior frontal gyrus, Right* | *Association* | *1.79* | *1.50* | 1.00 | 0.92 | 1.02 | 1.09 | 56.38 | 43.67 |
| *Inferior occipital gyrus, Left* | *Association* | *1.93* | *1.66* | 1.03 | 1.05 | 0.98 | 0.97 | 42.24 | 38.61 |
| *Amygdala, Left* | *Limbic* | *1.55* | *1.30* | 1.28 | 1.35 | 0.70 | 0.77 | 20.19 | 14.44 |
| *Parahippocampus gyrus, Left* | *Paralimbic* | *1.73* | *1.49* | 1.26 | 1.23 | 0.81 | 0.84 | 27.48 | 20.17 |
| *Calcarine Cortex, Right* | *Primary* | *1.83* | *1.59* | 1.00 | 0.96 | 1.01 | 1.05 | 51.38 | 41.61 |
| *Putamen, Left* | *Subcortical* | *1.77* | *1.54* | 1.06 | 1.01 | 0.95 | 1.00 | 45.52 | 34.28 |
| *Temporal pole (superior), Left* | *Paralimbic* | *1.68* | *1.45* | 1.06 | 1.13 | 0.97 | 0.90 | 32.76 | 36.67 |
| *Thalamus, Right* | *Subcortical* | *1.67* | *1.44* | 0.95 | 0.95 | 1.07 | 1.06 | 52.43 | 47.89 |
| *Fusiform gyrus, Left* | *Association* | *1.67* | *1.45* | 0.97 | 0.96 | 1.06 | 1.06 | 52.00 | 47.89 |
| *Thalamus, Left* | *Subcortical* | *1.70* | *1.48* | 0.97 | 1.00 | 1.04 | 1.01 | 46.29 | 44.78 |
| *Lingual gyrus, Right* | *Association* | *1.74* | *1.52* | 0.96 | 0.95 | 1.06 | 1.06 | 53.14 | 47.94 |
| *Inferior temporal gyrus, Left* | *Association* | *1.75* | *1.55* | 1.05 | 1.06 | 0.99 | 0.97 | 41.24 | 39.89 |
| *Orbitofrontal cortex (inferior), Left* | *Paralimbic* | *1.72* | *1.52* | 1.02 | 1.07 | 0.99 | 0.96 | 40.52 | 39.00 |
| *Superior frontal gyrus (medial)* | *Association* | *1.79* | *1.60* | 1.12 | 1.02 | 0.90 | 0.99 | 43.57 | 28.44 |
| *Insula, Left* | *Paralimbic* | *1.68* | *1.49* | 1.09 | 1.05 | 0.93 | 0.97 | 41.71 | 31.33 |
| *Supramarginal gyrus, Right* | *Association* | *1.81* | *1.62* | 1.07 | 1.01 | 0.95 | 1.01 | 45.81 | 34.33 |
| *Superior frontal gyrus (medial)* | *Association* | *1.78* | *1.59* | 1.17 | 1.03 | 0.88 | 0.99 | 43.71 | 27.44 |
| *Putamen, Right* | *Subcortical* | *1.73* | *1.54* | 1.04 | 1.00 | 0.97 | 1.01 | 46.43 | 36.56 |
| *Hippocampus, Right* | *Limbic* | *1.77* | *1.58* | 1.07 | 1.10 | 0.94 | 0.92 | 35.43 | 33.00 |
| *Fusiform gyrus, Right* | *Association* | *1.66* | *1.50* | 1.03 | 0.93 | 0.99 | 1.08 | 54.90 | 39.44 |
| *Orbitofrontal cortex (superior), Left* | *Paralimbic* | *1.73* | *1.57* | 1.02 | 1.05 | 1.00 | 0.97 | 42.38 | 40.56 |
| *Superior parietal gyrus, Left* | *Association* | *1.73* | *1.57* | 0.95 | 0.96 | 1.07 | 1.06 | 52.00 | 48.33 |
| *Middle temporal gyrus, Left* | *Association* | *1.65* | *1.48* | 0.96 | 0.98 | 1.05 | 1.03 | 49.00 | 45.28 |
| *Hippocampus, Left* | *Limbic* | *1.70* | *1.55* | 1.12 | 1.10 | 0.92 | 0.92 | 35.38 | 32.06 |
| *Middle occipital gyrus, Right* | *Association* | *1.77* | *1.61* | 0.97 | 1.01 | 1.04 | 1.01 | 46.14 | 45.33 |
| *Superior parietal gyrus, Right* | *Association* | *1.75* | *1.61* | 0.94 | 0.99 | 1.07 | 1.02 | 47.10 | 48.67 |
| *Temporal pole (superior), Right* | *Paralimbic* | *1.56* | *1.42* | 1.02 | 1.02 | 1.01 | 1.00 | 44.00 | 40.89 |
| *Inferior temporal gyrus, Right* | *Association* | *1.67* | *1.54* | 1.00 | 0.97 | 1.02 | 1.04 | 49.86 | 42.78 |
| *Middle Cingulate Gyrus, Right* | *Paralimbic* | *1.58* | *1.44* | 0.91 | 0.89 | 1.10 | 1.12 | 60.48 | 52.00 |
| *Calcarine Cortex, Left* | *Primary* | *1.74* | *1.61* | 0.96 | 1.00 | 1.04 | 1.01 | 46.81 | 45.11 |
| *Inferior frontal gyrus (triangular), Left* | *Association* | *1.75* | *1.62* | 1.03 | 1.03 | 0.98 | 0.98 | 43.24 | 37.33 |
| *Orbitofrontal cortex (superior), Right* | *Paralimbic* | *1.69* | *1.56* | 1.02 | 1.06 | 1.00 | 0.96 | 39.81 | 39.50 |
| *Supplementary motor area, Right* | *Association* | *1.63* | *1.50* | 0.92 | 0.93 | 1.09 | 1.08 | 55.19 | 51.56 |
| *Insula, Right* | *Paralimbic* | *1.47* | *1.59* | 1.13 | 1.10 | 0.90 | 0.95 | 39.71 | 29.22 |
| *Anterior Cingulate Gyrus, Left* | *Paralimbic* | *1.44* | *1.56* | 0.98 | 1.00 | 0.89 | 1.01 | 46.71 | 30.67 |
| *Orbitofrontal cortex (middle), Right* | *Paralimbic* | *1.81* | *1.70* | 1.09 | 1.12 | 0.94 | 0.92 | 35.76 | 33.22 |
| *Rectus gyrus, Left* | *Paralimbic* | *1.40* | *1.30* | 1.27 | 1.32 | 0.82 | 0.80 | 24.67 | 22.33 |
| *Superior temporal gyrus, Right* | *Association* | *1.59* | *1.49* | 0.95 | 0.97 | 1.07 | 1.04 | 50.14 | 48.28 |
| *Superior temporal gyrus, Left* | *Association* | *1.60* | *1.51* | 0.97 | 1.02 | 1.04 | 0.99 | 44.24 | 44.11 |
| *Orbitofrontal cortex (middle), Left* | *Paralimbic* | *1.71* | *1.62* | 1.04 | 1.09 | 0.97 | 0.94 | 38.19 | 36.28 |
| *Middle Cingulate Gyrus, Left* | *Paralimbic* | *1.54* | *1.45* | 0.89 | 0.90 | 1.13 | 1.12 | 59.90 | 55.33 |
| *Rolandic operculum, Right* | *Association* | *1.69* | *1.61* | 1.04 | 1.02 | 0.97 | 1.00 | 44.57 | 36.94 |
| *Precuneus, Left* | *Association* | *1.60* | *1.53* | 0.89 | 0.93 | 1.12 | 1.09 | 55.95 | 54.56 |
| *Middle Frontal Gyrus, Right* | *Association* | *1.62* | *1.55* | 0.97 | 0.93 | 1.04 | 1.08 | 54.71 | 45.00 |
| *Rolandic operculum, Left* | *Association* | *1.47* | *1.53* | 1.11 | 1.07 | 0.94 | 0.96 | 39.14 | 34.17 |
| *Precuneus, Right* | *Association* | *1.62* | *1.55* | 0.90 | 0.93 | 1.11 | 1.08 | 54.86 | 53.39 |
| *Middle occipital gyrus, Left* | *Association* | *1.67* | *1.62* | 0.92 | 1.00 | 1.09 | 1.02 | 48.05 | 51.22 |
| *Heschl's gyrus, Left* | *Primary* | *1.56* | *1.52* | 1.08 | 1.09 | 0.84 | 0.83 | 29.52 | 26.22 |
| Anterior Cingulate Gyrus, Right | Paralimbic | 0.98 | 1.56 | 1.24 | 1.02 | 0.85 | 1.00 | 45.71 | 26.17 |
| Rectus gyrus, Right | Paralimbic | 1.06 | 1.58 | 0.99 | 1.25 | 0.75 | 0.83 | 26.81 | 20.67 |
| Orbitofrontal cortex (medial), Right | Paralimbic | 1.83 | 1.43 | 1.18 | 1.17 | 0.85 | 0.89 | 32.29 | 23.78 |
| Postcentral gyrus, Left | Primary | 1.88 | 1.54 | 1.03 | 0.93 | 0.99 | 1.08 | 55.57 | 39.94 |
| Temporal pole (middle), Left | Paralimbic | 1.12 | 1.42 | 1.22 | 1.33 | 0.75 | 0.76 | 17.90 | 19.83 |
| Precentral gyrus, Left | Primary | 1.76 | 1.47 | 0.98 | 0.89 | 1.03 | 1.12 | 60.19 | 44.06 |
| Precentral gyrus, Right | Primary | 1.78 | 1.49 | 0.98 | 0.90 | 1.04 | 1.12 | 59.67 | 45.39 |
| Amygdala, Right | Limbic | 1.25 | 1.52 | 1.16 | 1.28 | 0.77 | 0.80 | 22.33 | 19.33 |
| Lingual gyrus, Left | Association | 1.78 | 1.51 | 0.96 | 0.97 | 1.05 | 1.04 | 50.14 | 46.83 |
| Angular Gyrus, Left | Association | 1.29 | 1.56 | 0.95 | 1.19 | 0.79 | 0.87 | 29.95 | 23.39 |
| Orbitofrontal cortex medial), Left | Paralimbic | 1.86 | 1.60 | 1.17 | 1.12 | 0.87 | 0.90 | 33.48 | 26.72 |
| Posterior Cingulate Gyrus, Right | Paralimbic | 1.85 | 1.62 | 1.08 | 1.05 | 0.94 | 0.97 | 41.71 | 33.11 |
| Temporal pole (middle), Right | Paralimbic | 1.36 | 1.59 | 1.03 | 1.16 | 0.85 | 0.89 | 32.62 | 27.39 |
| Caudate, Right | Subcortical | 1.41 | 1.21 | 1.00 | 1.22 | 0.89 | 0.85 | 29.38 | 33.00 |
| Paracentral lobule, Right | Association | 1.79 | 1.58 | 0.98 | 1.02 | 1.04 | 1.00 | 45.57 | 46.44 |
| Cuneus, Right | Association | 1.79 | 1.59 | 0.98 | 0.96 | 1.04 | 1.06 | 52.48 | 45.33 |
| Pallidum, Right | Subcortical | 1.80 | 1.60 | 1.09 | 1.01 | 0.93 | 1.00 | 44.52 | 31.78 |
| Superior frontal gyrus, Left | Association | 1.71 | 1.52 | 0.98 | 0.94 | 1.03 | 1.07 | 53.38 | 43.44 |
| Inferior occipital gyrus, Right | Association | 2.00 | 1.81 | 1.08 | 1.15 | 0.93 | 0.89 | 32.14 | 32.67 |
| Postcentral gyrus, Right | Primary | 1.75 | 1.56 | 0.98 | 0.92 | 1.04 | 1.10 | 57.00 | 44.61 |
| Cuneus, Left | Association | 1.83 | 1.65 | 1.00 | 1.00 | 1.01 | 1.01 | 46.48 | 41.94 |
| Inferior frontal gyrus (opercular), Left | Association | 1.80 | 1.64 | 1.10 | 1.04 | 0.92 | 0.97 | 42.10 | 30.89 |
| Parahippocampus gyrus, Right | Paralimbic | 1.68 | 1.53 | 1.16 | 1.09 | 0.87 | 0.95 | 39.76 | 25.50 |
| Inferior frontal gyrus (triangular), Right | Association | 1.73 | 1.59 | 1.02 | 0.96 | 1.00 | 1.04 | 50.71 | 40.00 |
| Inferior frontal gyrus (opercular), Right | Association | 1.72 | 1.57 | 1.01 | 0.96 | 1.00 | 1.05 | 51.33 | 40.22 |
| Superior occipital gyrus, Right | Association | 1.82 | 1.68 | 0.97 | 1.00 | 1.05 | 1.02 | 47.81 | 46.28 |
| Supplementary motor area, Left | Association | 1.68 | 1.53 | 0.96 | 0.96 | 1.06 | 1.05 | 51.52 | 47.44 |
| Olfactory, Left | Limbic | 1.44 | 1.58 | 1.12 | 1.21 | 0.79 | 0.84 | 27.33 | 20.44 |
| Orbitofrontal cortex (inferior), Right | Paralimbic | 1.64 | 1.50 | 0.97 | 0.96 | 1.04 | 1.05 | 51.05 | 44.67 |
| Middle temporal gyrus, Right | Association | 1.59 | 1.46 | 0.92 | 0.93 | 1.09 | 1.08 | 55.10 | 50.33 |
| Supramarginal gyrus, Left | Association | 1.80 | 1.67 | 1.06 | 1.02 | 0.96 | 0.99 | 43.71 | 34.61 |
| Middle Frontal Gyrus, Left | Association | 1.68 | 1.54 | 0.96 | 0.93 | 1.05 | 1.08 | 55.71 | 46.11 |
| Pallidum, Left | Subcortical | 1.74 | 1.62 | 0.99 | 1.06 | 1.02 | 0.96 | 39.81 | 42.33 |
| Superior occipital gyrus, Left | Association | 1.80 | 1.70 | 0.96 | 1.02 | 1.05 | 1.00 | 45.67 | 47.06 |
| Olfactory, Right | Limbic | 1.30 | 1.21 | 1.18 | 1.22 | 0.76 | 0.84 | 26.62 | 19.00 |
| Inferior parietal lobule, Left | Association | 1.71 | 1.62 | 0.99 | 0.99 | 1.03 | 1.02 | 47.43 | 43.28 |
| Angular Gyrus, Right | Association | 1.59 | 1.64 | 1.02 | 1.07 | 0.86 | 0.96 | 40.90 | 27.22 |
| Caudate, Left | Subcortical | 1.44 | 1.45 | 1.03 | 1.19 | 0.86 | 0.87 | 30.86 | 29.61 |
